# Positive Stress Electrocardiography in Patients With Non-obstructive Coronary Disease

**DOI:** 10.7759/cureus.35549

**Published:** 2023-02-27

**Authors:** Niya E Semerdzhieva, Stefan V Denchev

**Affiliations:** 1 Emergency Department, University Hospital "St. Anna", Sofia, BGR; 2 Cardiology Department, Medical Centre "Mediva", Sofia, BGR

**Keywords:** corrected timi frame count, left ventricular hypertrphy, slow coronary flow phenomenon, non-obstructive coronary disease, exercise stress electrocardiography

## Abstract

Introduction

The episodes of myocardial ischemia in patients with non-obstructive coronary disease are extremely variable in provoking factors and presentation.

Purpose

We investigated the significance of coronary blood flow velocity and epicardial diameter as correlates of a positive electrocardiographic exercise stress test (ExECG) in hospitalized patients with unstable angina and non-obstructive coronary artery disease.

Methods

The study was a single-center cohort retrospective. ExECG was performed and analyzed in a group of 79 patients with non-obstructive coronary disease (coronary stenoses < 50%). Thirty-one percent of the patients (n=25) were diagnosed with slow coronary flow phenomenon, SCFP; 40.5% (n=32) - patients with hypertensive disease, left ventricular hypertrophy (LVH), and slow epicardial flow; 27.8% (n=22) with hypertension, left ventricular hypertrophy and normal coronary flow. The patients were hospitalized in University Hospital “Alexandrovska,” Sofia in the period 2006-2008.

Results

The frequency of positive ExECG is increased as a trend was associated with smaller epicardial diameters and pronounced delay in epicardial coronary flow. In the subgroup with SCFP, the risk for a positive ExECG test was determined by slower coronary flow (36.5±7.7 frames vs. 30.3±4.4 frames, p=0.044) and borderline significant by epicardial lumen diameters (3.3±0.8 mm vs. 4.1±1.0 mm, p=0.051) and greater myocardial mass (92.8±12.6 g/m^2^ vs. 82.9±8.6 g/m^2^, p=0.054). In cases of left ventricular hypertrophy, which included both patients with the normal and slow epicardial flow, there were no statistically significant correlates of an abnormal exercise stress ECG test.

Conclusions

In patients with non-obstructive coronary atherosclerosis and predominantly slow epicardial coronary flow, the provoking of ischemia at an electrocardiographic exercise stress test is associated with the lower epicardial flow velocity at rest and with the smaller epicardial diameter. In SCFP, the risk for an abnormal stress test is determined by slower coronary flow, smaller epicardial lumen diameter, and greater myocardial mass. The presence and size of the plaque burden are not associated with a greater risk of a positive ExECG in these patients.

## Introduction

The episodes of myocardial ischemia in patients with non-obstructive coronary disease are extremely variable in provoking factors and presentation [1,2]. The pathogenesis of cardiac ischemia in this heterogeneous population remains poorly understood. The provocation of cardiac ischemia. has shown a strong positive relationship between smaller coronary artery diameters [3], and increased microvascular resistance under stress [4,5].

Purpose

We investigated the significance of coronary blood flow velocity and epicardial diameter as correlates of a positive electrocardiographic stress test in hospitalized patients with unstable angina and non-obstructive coronary artery disease.

## Materials and methods

The current research involved a single-center cohort retrospective study. A total of 79 patients with non-obstructive coronary disease (coronary stenoses < 50%) were analyzed: 31.6% (n = 25) with slow coronary flow phenomenon, SCFP; 40.5% (n = 32) - patients with the hypertensive disease, left ventricular hypertrophy (LVH) and slow epicardial flow (SF_LVH_); 27.8% (n = 22) with hypertension, left ventricular hypertrophy and normal coronary flow (NF_LVH_). The patients were hospitalized at University Hospital “Alexandrovska,” Sofia in the period 2006-2008. A symptom-limited electrocardiographic stress exercise test (ExECG) was performed on all patients using the Bruce protocol [6]. The criteria for positive ExECGs were the occurrence of angina or ST depression (ST elevation) equal to or greater than 0.1 mV (1 mm) 0.08 sec after the J-point [7]. The coronary flow impairment was determined using the corrected Thrombolysis in the Myocardial Infarction frame count (cTFC) method [8].^ ^For each patient with coronary stenoses detected at conventional coronary angiography (CAG), Gensini's angiographic score was calculated [9].^ ^Left ventricular volumes, ejection fraction, and myocardial mass were determined by two-dimensional cardiac ultrasound using standard methods [10]. Left ventricular hypertrophy was defined as left ventricular free wall or septum thickness equal to or greater than 12 mm and myocardial mass greater than 95 g/m^2^ for female patients or 115 g/m^2^ for male patients. The myocardial mass was calculated according to the equation:

LV myocardial mass = 0.8x[1.04x(EDD+IVSd+LVPWd)^3^ - (EDD)^3^]+0.6

where EDD stands for end-diastolic dimension, given in centimeters; IVSd is interventricular septum thickness at end-diastole in centimeters; LVPWd means the thickness of the posterior wall of the left ventricle at end-diastole in centimeters; 1.04 - is the heart muscle density in gram/cubic meter (g/cm³). The left ventricular mass index (LVMI) is derived by dividing LV myocardial mass by the body surface area of the patient.

Exclusion criteria for the study were: prior thrombolytic therapy for acute myocardial infarction or prior percutaneous coronary intervention; ejection fraction < 50%; moderate/severe valvular disease; coronary ectasia, coronary aneurysms, myocardial bridges; use of non-selective β-blocker and β-blocker with vasodilating properties, bundle branch block (left and right), ST depression >0.5 mm, negative and biphasic T waves and paced rhythm on the resting ECG. Patients with coronary stenoses < 40% and without left ventricular hypertrophy from the slow coronary flow phenomenon subgroup.

Statistical analysis was performed with the SPSS program, version 28.0.1.0, by applying distribution estimation methods (Shapiro-Wilk and Kolmogorov-Smirnov methods), parametric (Student's t-test, ANOVA) and nonparametric methods (χ^2^-test; Mann-Whitney test; Kruskal-Wallis methods) depending on the type of the variable (categorical or quantitative) and its distribution (normal or not). Variables are presented as mean ± standard deviation (quantitative variables) or as count or percentage (categorical). Relationships of a significance level (α) less than 0.05 were considered statistically significant.

## Results

Baseline characteristics

**Figure 1:**
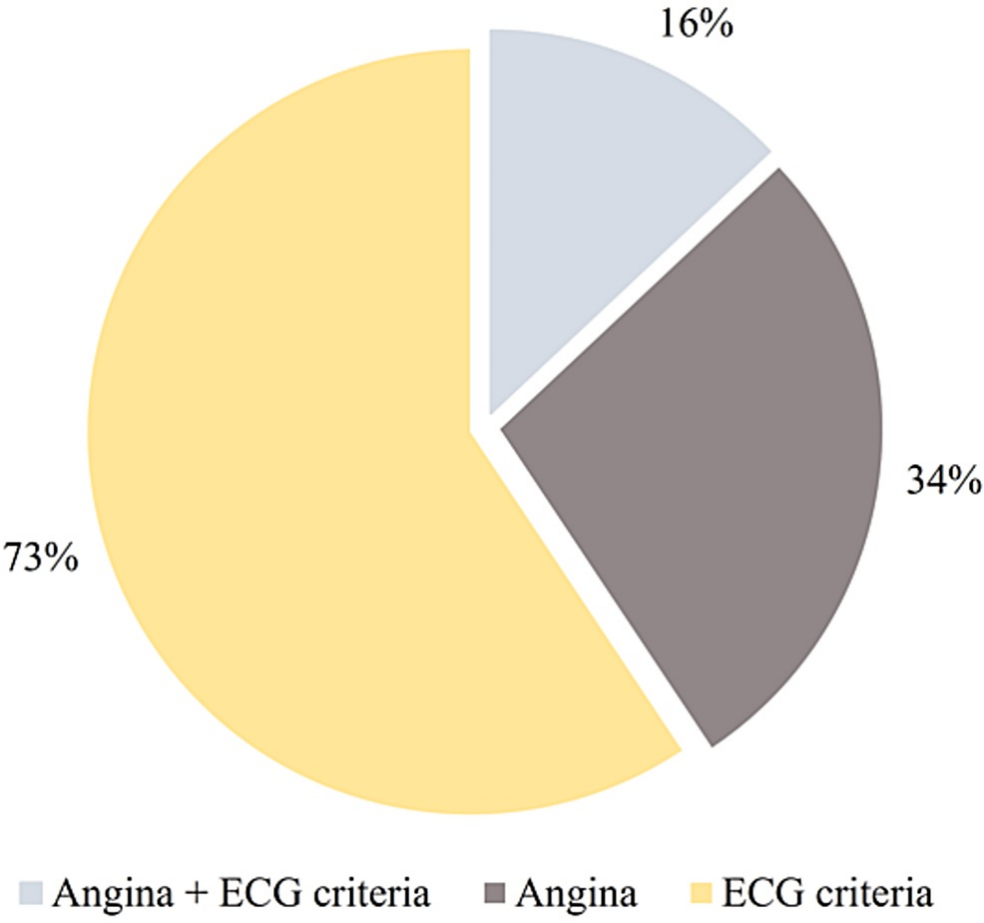
Incidence of angina and/or ECG abnormalities at exercise electrocardiography ECG criteria - electrocardiographic criteria for positive test

In 16% of patients, angina, and ECG changes were induced during ExECG (Figure 1).

Coronary stenoses of up to 40% were found in most patients, and isolated 50% stenoses were present in 17% (n = 3) of the patients. Less than half (40%) of the patient population had two or three coronary plaques. In 7.9%, delayed epicardial blood flow was present in the three coronary arteries. One patient (0.7%) displayed transient ST elevation during stress; in two others a coronary spasm was induced by the diagnostic catheter at angiography (Table 1).

**Table 1:** Characteristics of the study group SCFP – slow coronary flow phenomenon; SF_LVH_ + NF_LVH _– patients with left ventricular hypertrophy and slow plus patients with LH hypertrophy and normal epicardial coronary flow; ExECG – stress electrocardiography; ACE-I – an inhibitor of angiotensin-converting enzyme;  ARB – angiotensin receptor blocker; CCB – calcium channel blocker; MET – metabolic equivalents; SBPmax – systolic blood pressure at maximal exercise; HRmax – heart rate at maximal exercise; Time – time interval of exercise; Depic – epicardial coronary lumen diameter; cTFC – corrected Thrombolysis in Myocardial Infarction frame count;  EF – ejection fraction; EDVI/ESVI –  index of the left ventricular end-diastolic/end-systolic volume; LVMI – left ventricular myocardial mass index

Variables	Patients	SCFP	SF_LVH_+NF_LVH_	P-value
	n %	n %	n %	
Men/Women	22/57 (27.8/72.2)	10/13 (45.4/54.5)	12/44 (21.4/78.6)	0.183
Positive ExECG	45 (57)	17 (68)	28 (53.9)	0.324
Hypertension	69 (87.3)	20 (76.9)	49 (92.5)	NS
Dyslipidemia	48 (73.8)	12 (70.6)	36 (75)	0.722
Diabetes mellitus	12 (15.2)	4 (15.4)	8 (15.1)	1.000
Smoking	10 (12.7)	1 (3.8)	9 (17)	0.153
ACE-I/ARB	56 (70.9)	18 (69.2)	38 (71.7)	1.000
Β- blocker	38 (51.3)	14 (53.8)	24 (46.2)	
Β- blocker+CCB+nitrate	19 (24.3)	1 (3.8)	18 (71.7)	NS
Β- blocker+ nitrate	19 (24.3)	5 (19.2)	5 (9.6)	
No vasoactive therapy	2 (2.6)	1 (3.8)	1 (1.9)	
	Mean±SD	Mean±SD	Mean ±SD	P-value
Age, years	58.7±8.5	56±8.2	60±8.4	0,051
METs	10.8±17.7	9.5±2.1	11.3±21.3	0.735
Time, min	4.5±1.5	5.1±1.4	4.5±2	0.636
SBPmax, mmHg	151.9±24.3	151.5±19.4	152.9±26.7	0.919
HRmax, min	124.2±17.2	135.1±21.8	120.8±13.4	0.016
Depic, mm	3.6±0.8	3.6±0.9	3.6±1.8	0.777
cTFC, frames	33.3±15.3	34.6±7.4	32.6±17.9	0.591
Coronary stenoses 15-50%	17 (21.5)	5 (22.7)	12 (20)	NS
Gensini score	0.93±2	0.98±2.52	0.87±1.77	0.826
EF, %	67.6±6.8	67.6±5.4	67.6±7.6	0.971
EDVI ml/m^2^	62.2±13.8	56.3±10.8	65.3±14.3	0.006
ESVI ml/m^2^	20.4±6.3	18.3±4.2	21.6±6.9	0.036
LVMI, g/m^2^	115.4±26.1	89.9±12.3	128.4±21.1	0.001

The arterial blood pressure (BP), diabetes mellitus and dyslipidemia were not optimally controlled by the therapy in 7.9%, 8% and 19% of the patients, respectively. The mean blood pressure, plasma glucose and lipid levels in the studied group at the hospital admission were systolic BP - 131.5 ± 19.5 mmHg, diastolic BP - 81.2 ±12.2 mmHg, glucose - 5.4±1.9 mmol/L; total cholesterol - 5.2±1.1 mmo/L, high density lipoproteins (HDL) - 1.3±0.2 mmol/L, low density lipoproteins (LDL) - 3.1±0.9 mmol/L and triglycerides (TG) - 1.7±1.0 mmol/L.

Stress ECG, coronary flow, and epicardial diameter

The presence and size of the plaque burden are not associated with a greater risk of a positive ExECG in the studied group (Table 2).

**Table 2:** Correlates of positive ExECG SCFP – slow coronary flow phenomenon; SF_LVH_+NF_LVH_ – patients with left ventricular hypertrophy; ExECG – stress electrocardiography; Depic – epicardial coronary lumen diameter; cTFC – corrected thrombolysis in myocardial infarction frame count; EF – ejection fraction; EDVI/ESVI - index of the left ventricular end-diastolic/end-systolic volume; LVMI – left ventricular myocardial mass index

Variables	Negative ExECG	Positive ExECG	P-value
Patients	n = 43	n = 36	
Depic	3.8±1	3.5±0.7	0.076
cTFC	29.7±13.4	35.7±16.3	0.079
Gensisni score	0.74±1.56	1.06±2.31	0.495
EDVI	62.8±14.1	61.3±13.6	0.742
ESVI	20.2±6.6	20.4±6	0.754
EF	67.8±8.1	76.4±5.7	0.803
LVMI	118.4±30.2	112.6±22.7	0.347
SCFP	n = 18	n = 8	P-value
Depic	4.1±1	3.3±0.8	0.051
cTFC	30.2±4.4	36.5±7.7	0.044
EDVI	59±11	55.1±10.8	0.204
ESVI	19.2±5.3	18±3.7	0.248
EF	66.5±4.4	68.1±5.8	0.508
LVMI	82.8±8.6	92.8±12.6	0.054
SF_LVH_+NF_LVH_	n = 25	n = 28	P-value
Depic	3.6±0.9	3.5±0.5	0.550
cTFC	29.5±15.3	35.4±15.3	0.569
EDVI	64.2±15	66.3±13.8	0.613
ESVI	20.5±7.1	22.5±6.8	0.332
EF	68.3±9	67±5.7	0.550
LVMI	130.2±25	126.8±17.2	0.569

Subgroup analysis

In the subgroup with SCFP, the risk for a positive stress ECG test was determined by slower coronary flow (36.5±7.7 frames vs. 30.3±4.4 frames, p=0.044) and borderline significant by epicardial lumen diameters (3, 3 ±0.8 mm vs. 4.1±1.0 mm, p=0.051) and greater myocardial mass (92.8±12.6 g/m^2^ vs. 82.9±8.6 g/m^2^, p=0.054). In cases of left ventricular hypertrophy, which included both patients with normal and slow epicardial blood flow, there were no statistically significant correlates of an abnormal ExECG test.

There were no differences in characteristics (myocardial mass, epicardial blood flow delay, etc.) between patients with minimal versus maximal epicardial diameter at analysis. Maximum delay of coronary flow (maximum cTFC) in the entire studied group was found more often in patients with a myocardial mass of the middle (second and third) quartile (112.8±20.9 g/m^2^ vs. 135.3±21.6 g/m^2^, p<0.001). The age of patients with the slowest did not differ from that of patients with the intact epicardial flow (61.6±9 years vs. 59.3±8.3 years, p=0.183). The peak heart rate in patients with left ventricular hypertrophy was significantly lower compared to SCFP (Table 2).

The frequency of positive ExECGs in the patients on beta-blocker (BB) as only vasoactive angina medication was higher (although insignificantly) than the other two vasoactive therapeutic combinations (positive ExECG - BB vs BB+calcium blocker±nitrate vs BB+nitrate - 68.3% [n=28] vs 14.6% [n=6] vs 17.1% [n=7], p-NS).

The analysis of the patients with the slow coronary flow in the anterior descending branch of the left coronary artery (n=70) attenuated the relationship of coronary flow and positive ExECG (cTFC - 29.4±10 frames vs. 33.7±10.5 frames, p=0.085). The rest of the results became insignificant in the whole group (3.7±1.0 mm vs. 3.5±0.7mm, p=0.213; LVMI - 116.9±29.5 g/m^2^ vs. 111±21.7 g/m^2^, p=0.350) and among SCFP patients (3.8±1.1 mm vs. 3.4±0.9 mm, p=0.238; cTFC - 34.2±5.3 frames vs 37.5±7.7 frames, p=0.278; myocardial mass - 85.3±9.8 g/m^2^ vs. 93.3± 12.3 g/m^2^, p=0.149).

## Discussion

The results of our study show that the tendency for a positive exercise stress test in a heterogeneous group with non-obstructive coronary disease is related to the impairment in epicardial flow at rest. In previous studies, pathological exercise tests were conducted that demonstrated a higher prevalence of exercise-induced radionuclide abnormalities in patients with non-obstructive coronary disease [1,11], and the exercise-induced functional and perfusional abnormalities were found to be simultaneously present most frequently in coronary territories supplied by vessels with delayed run-off [11].

The trend for abnormal ExECG in our non-obstructive coronary disease population with various myocardial masses and impairment in epicardial coronary flow was due to a significant relationship in the subgroup of patients with SCFP. SCFP, also called “unstable microvascular angina” [12],^ ^presents typically as resting or mixed angina (at rest and exertion). Fineschi et al. reported increased coronary arterial tone at rest and Beltrame et al. gathered evidence for low myocardial blood oxygen saturation at rest in the SCFP [12,13].^ ^A delay in epicardial coronary flow (increased cTFC) was previously demonstrated in a patient population with coronary micro- and macrovascular spasms [14]. The response to stress in SCFP is typically variable [15]. Microvascular dysfunction (impaired coronary response to vasodilating drugs) was a proposed pathologic mechanism of angina in SCFP [3].

Also on that note, the variability in stress exercise test results in the non-obstructive coronary disease population is often explained by coronary arterial micro-or macrovascular spasms upon exertion. The coronary flow reserve may remain normal in patients with focal coronary spasms, in contrast to those with diffuse vasoconstriction [16].^ ^Altogether, stress ECG changes are usually recorded at the time of stress ECG testing in all patients with a tendency to vasoconstriction [17].^ ^A tendency for spasms in the distal part of the coronary artery is possibly the rationale for the risk of stress ischemia in our study group. Firstly, in the presented study, ischemic stress ECG changes did not indicate the main blood supply area, but remote myocardial areas. They corresponded to the distal part of the coronary artery with the most delayed blood flow. Secondly, the magnitude of the plaque atherosclerotic burden was not associated with a greater risk for a positive ExECG in our study. A likely reason is the high incidence of atherosclerotic irregularities in patients with up to intermediate epicardial stenoses [18].^ ^Each plaque, depending mainly on the degree of infiltration with macrophages, can be a source of angiotensin-converting enzyme and high local levels of angiotensin II, therefore creating a risk of coronary spasm under stress [19]. The therapy with β-blocker as the only vasoactive angina medication was higher (although insignificantly) than the other two vasoactive therapeutic combinations. Calcium channel blockers (but not β-blockers) suppress episodes of microvascular vasospasm [20].

An important result of the study is the positive relationship between higher myocardial mass within normal limits and the risk of a positive stress ECG in patients with SCFP. Importantly, it was observed even after the exclusion of patients with myocardial remodeling (increased left ventricular cavity dimensions, normal left ventricular wall thickness, and abnormally increased calculated myocardial mass) from the SCFP group. The association of the small surface area of ​​the coronary lumens and the limitation in coronary blood flow reserve in a given myocardial area has been confirmed in a computer tomography-based assessment of fractional blood flow reserve in patients without coronary atherosclerosis [21]. Small coronary diameters in SCFP have been reported in histologic studies [3]. Data from such studies give a partial explanation of the relationship of epicardial diameter with myocardial ischemia occurrence in patients with SCFP.

Our results show that when the patients with LV hypertrophy and non-obstructive coronary disease are analyzed along with SCFP patients, the relationship of the positive ExECGs with epicardial lumen diameters and coronary flow weakens. The size of the coronary diameters in our study did not differ in relation to the myocardial mass and other characteristics of the patients. The epicardial flow, a correlate of positive ExECG, shows a complex but significant relationship with the myocardial mass. Various mechanisms of myocardial ischemia in patients with non-obstructive coronary disease have been reported [22].^ ^In hypertrophy, higher levels of biologically active molecules with vasodilating properties in the systemic circulation have been previously demonstrated [23].^ ^These components are synthesized in the endothelium under the influence of increased ventricular pressures and arterial shear stress. Studies suggested their involvement in positive vascular remodeling [23].^ ^The result is positive epicardial artery remodeling and consequent normal or increased coronary blood flow at rest. However, this compensatory mechanism is often not sufficient for the normal functioning of the hypertrophied myocardium during exercise [24].

 Limitations

Major limitations of the study are the small study sample and hence this requires further validation using a larger population; the comparison of coronary flow at rest with variables (symptoms, ECG changes) at exercise; the performance of CAG and ExECG without stopping the angina therapy; and also, the lack of subdiagnosing of the patients as those having a coronary vasospastic coronary disease or microvascular angina using specific currently recommended methods [20,25].

## Conclusions

In patients with non-obstructive coronary atherosclerosis and predominantly slow epicardial coronary flow, the provoking of ischemia at an electrocardiographic exercise stress test is associated with the lower epicardial flow velocity at rest and with the smaller epicardial diameter. In SCFP, the risk for an abnormal stress test is determined by slower coronary flow, smaller epicardial lumen diameter, and greater myocardial mass. The presence and size of the plaque burden are not associated with a greater risk of a positive ExECG in these patients.
